# Plasmon-Assisted Audio Recording

**DOI:** 10.1038/srep09125

**Published:** 2015-03-16

**Authors:** Hao Chen, Abdul M. Bhuiya, Qing Ding, Kimani C. Toussaint, Jr.

**Affiliations:** 1 Department of Mechanical Science and Engineering, University of Illinois Urbana–Champaign, Urbana, Illinois 61801, USA; 2 Department of Electrical and Computer Engineering, University of Illinois Urbana–Champaign, Urbana, Illinois 61801, USA; 3 Visiting Associate Professor, Department of Mechanical Engineering, Massachusetts Institute of Technology, Cambridge, Massachusetts 02139, USA

## Abstract

We present the first demonstration of the recording of optically encoded audio onto a plasmonic nanostructure. Analogous to the “optical sound” approach used in the early twentieth century to store sound on photographic film, we show that arrays of gold, pillar-supported bowtie nanoantennas could be used in a similar fashion to store sound information that is transferred via an amplitude modulated optical signal to the near field of an optical microscope. Retrieval of the audio information is achieved using standard imaging optics. We demonstrate that the sound information can be stored either as time-varying waveforms or in the frequency domain as the corresponding amplitude and phase spectra. A “plasmonic musical keyboard” comprising of 8 basic musical notes is constructed and used to play a short song. For comparison, we employ the correlation coefficient, which reveals that original and retrieved sound files are similar with maximum and minimum values of 0.995 and 0.342, respectively. We also show that the pBNAs could be used for basic signal processing by ablating unwanted frequency components on the nanostructure thereby enabling physical notch filtering of these components. Our work introduces a new application domain for plasmonic nanoantennas and experimentally verifies their potential for information processing.

In recent years, applications of plasmonic nanoantennas have focused on optical trapping^1^,^2^, basic studies in thermoplasmonics^3^,^4^,^5^, solar energy harvesting^6^ and biosensing^7^. The particularly attractive feature of metal nanoantennas is their ability to concentrate light into sub-wavelength regions with local field enhancements as high as 10^4^ 8,9. Generally, nanoantennas are fabricated bound to a dielectric substrate, with their geometry and functionality remaining fixed after fabrication. Still, there has been a recent push to place the nanoantennas on pillars, thereby elevating them above the substrate, in order to increase the field/sensitivity enhancement for sensor applications^10^,^11^,^12^. Indeed, it has been shown that an array of Si pillar-supported nanoantennas could enhance the signal for surface enhanced Raman scattering^10^. Even more recently, we have demonstrated that Au bowtie nanoantenna arrays on glass pillars exhibit not only significant field enhancement, but their thermal properties become much more sensitive to the input optical intensity. Specifically, we showed that the radius of curvature of the nano triangles that comprise these pillar-supported bowtie nanoantennas (pBNAs) can be spatially tuned via local optical-induced heating such that up to 100-nm shifts in the plasmonic resonance response can be obtained^13^. Interestingly, this approach resulted in a photographic film effect, whereby these nanostructures can record the near-field optical intensity at low input power densities in real time, and thus be used to create textured plasmonic surfaces for optical trapping^13^.

In this Letter, we demonstrate that an array of Au pBNAs can be used to store optically encoded audio information for subsequent retrieval and playback. To our knowledge, this is the first demonstration of a nonmagnetic, plasmonic nanostructure recording audio information. Our approach is analogous to the method of “optical sound”, which was developed circa 1920s as part of the effort to make “talking” motion pictures. Although there were variations of this process, they all shared the same basic principle. An audio pickup, e.g., a microphone, electrically modulates a lamp source. Variations in the intensity of the light source is encoded on semi-transparent photographic film (e.g., as variation in area) as the film is spatially translated. Decoding this information is achieved by illuminating the film with the same light source and picking up the changes in the light transmission on an optical detector, which in turn may be connected to speakers. In the work that we present here, the pBNAs serve the role of the photographic film which we can encode with audio information via direct laser writing in an optical microscope. We show that this process of audio recording is enabled by modulating a pair of galvonometer-driven, laser-scan mirrors with our desired sound file, thereby amplitude modulating the laser beam used to record the audio file on the plasmonic film. In addition, since we use an optical microscope for recording, we choose to retrieve this audio information simply by imaging the region of the exposed pBNAs using dark-field microscopy and subsequently extracting the signal during post processing. We show that this recording process can be done either in the time or frequency domain, the latter of which allows physical filtering of unwanted frequency components.

The experimental setup used for audio recording and reading is detailed in Fig. 1a. A tunable Ti:Sapphire laser is used to produce pulses at center wavelength of 780 nm and temporal width of 100 femtosecond. The pulses are polarized along the long axis of the pBNAs. A high-speed scanning galvanometer mirror positioning system (Thorlabs GVS012) directs the pulses into the microscope system for optical beam steering. In the upright orientation, the pBNAs are illuminated from the substrate side. The audio recording and retrieval system are built around a customized inverted microscope (Olympus IX81) with a 0.6 numerical aperture (NA), collar-adjustable microscope objective (Olympus LUCPlanFLN 40×) used for illumination, which produces an approximately Gaussian intensity distribution with a full width at half maximum of 790 nm. On the other side of the sample, a 0.9 NA, microscope objective (Olympus MPlan LFN 100×) is used for dark-field imaging. A halogen lamp, white light source (Dolan Jenner, 190) is used to image the pBNAs onto a CMOS color camera (Thorlabs, DCC1645C), which is preceded by a laser-blocking band-pass filter.

The 80 × 80-*μ*m^2^ area of the plasmonic film comprises of a 425 × 425 nm-spaced array of pBNAs that are fabricated on top of a 25-nm thick ITO layer and a 400-*μ*m thick SiO_2_ substrate shown in Fig. 1b. Gold BNAs are sandwiched between an 8-nm thick Ni protective top layer and a 5-nm thick Cr adhesion bottom layer. The fabricated pBNAs have, on average, a 35-nm gap spacing and pillars with a height of 500 nm. The pBNAs are illuminated off-resonance at a wavelength of 780 nm. The functionality of plasmonic film for audio recording is derived from photothermally induced morphological changes in the Au particles. In order to observe visible changes with high contrast, 50 mW average power is used for all experiments. The optical response of the unexposed and exposed region of the film is assessed by measuring the spectral reflectance, shown in Fig. 1c. A 60-nm spectral shift is observed for the exposed area relative to the unexposed area.

Galvo operation for audio recording is programmed in Labview (National Instruments Corporation). The galvo driver is connected to a DAQ board (NI USB-6221) with the position of mirrors controlled by the output voltage. An audio signal stored in a computer is converted by Labview to an amplitude-varying voltage that drives the galvo mirrors. With a given sampling frequency of the original audio waveform, the number of points required to record the audio is determined. Given the 0.6 NA illumination objective, and the fact that the mechanical resolution of the galvo scan angle is 14 *μ*rad, the distance between the sampling points on the plasmonic film is 45 nm^14^. The normalized amplitude of the recorded audio is set as 6.5 *μ*m. By transferring the audio information into voltages, waveform recording is enabled by laterally scanning the laser spot on the plasmonic film.

Dark-field imaging is used to record the image of the written waveform on the plasmonic film. In principle, bright-field imaging can also be used, but dark-field imaging provides high contrast images that facilitate post processing. An RGB image is recorded by the color camera with the image background in red and the ~790-nm thick waveform in green. In the audio retrieving process, only the green element value is extracted from the RGB image. Note that a gradual color change in image pixels from green to red is observed at the edge of the waveform. However, through a standard edge detection approach^15^, a single value can be determined for each lateral position.

Figure 2 provides a top view, scanning electron microscopy (SEM) image of the plasmonic film after a vertical-line illumination pattern (overlayed in red on the SEM). Visible morphological changes of the radius of curvature of each Au triangular tip, which in turn modifies the gap size, are observed. Not surprisingly, the gradual shape change along the horizontal direction in the image is indicative of the intensity gradient distribution from a Gaussian beam. This results in a direct color change on the pBNAs. As shown in Fig. 2b, a line plot of the change in gap size versus transverse distance fits well with an intensity Gaussian profile of a 780-nm wavelength laser beam focused by a 0.6 NA objective. We employ basic image processing to convert the 2D pBNA image to a 1D time-varying audio signal.

Figure 3 shows the results of writing 8 distinct musical notes onto the pBNAs. In this case, from Fig. 3(a–h), we successfully record middle C (C_4_), D, E, F, G, A, B and tenor C (C_5_) on the plasmonic film. As shown in the actual recorded images, each note is a single harmonic and the overall range of frequencies is from 261.63 Hz to 523.25 Hz. In our recording process, since each note is generated in a digital format with a standard sampling frequency of 44.1 kHz, one second of audio on the film corresponds to 44,100 illuminated points in a given segment. Similar to the musical keys on a keyboard, the notes can be played in a specified order to generate a desired sound. We demonstrate this type of “plasmonic keyboard” by taking the 8 recorded musical notes, and in the post-processing, arranging them to construct a short song (see Supplementary audio 1 for ‘twinkle, twinkle little star’).

To analyze the quality of the retrieved waveform read from the plasmonic film, we use the correlation coefficient *ρ_X,Y_* = *cov*(*X,Y*)/(*σ_X_*·*σ_Y_*) to assess the quality of retrieval audio^16^, where, *X* and *Y* represent the original and retrieved signals, respectively, *cov* represents the covariance operation, and *σ_X_* and *σ_Y_* are the standard deviation of the corresponding original and retrieved signals. The correlation coefficients for the retrieved 8 notes in Fig. 3(a–f) are listed in Table 1. With an increase of the tone, a waveform with higher frequency oscillations is recorded within a unit area that causes aliasing. Thus, a decrease of the correlation coefficient is observed from C_4_ to C_5_.

In a similar fashion, we next demonstrate the effectiveness of our approach in recording vocal (human voice) information. Here, a 470 ms recording of “hello” is recorded (See Supplementary audio 2 and 3 for the original and retrieved audio files, respectively). Figure 4a shows the original time-varying signal. To inspect more closely, we expand a short segment of the audio file (~40 ms in duration) as shown in Fig. 4b. We compare the corresponding segment in Fig. 4c from the retrieved audio profile in Fig. 4d. Note that for improved quality, the spatial extent on the plasmonic film of this audio file is ~900 *μ*m, which cannot fit into the total area of a single pBNA array (80 × 80 *μ*m^2^). Thus, we use 7 additional arrays to stitch together the audio signal. However, due to the possible mismatch at the boundaries between the arrays, perfectly aligning the various pieces of the audio signal proves challenging, and we obtain a relatively low correlation coefficient of 0.342. Still, as proof-of-concept, it is clear from the audio that the plasmonic film can store audio (voice) information, as well.

To evaluate the capacity of the plasmonic film, we estimate the area required to store one second of audio information. For a standard recording process, the maximum amplitude is set as 13 *μ*m and one second of audio is stored with a length requirement of 1962.2 *μ*m. Therefore, an area of 0.0255 mm^2^ is required on our plasmonic film for one second of audio storage. In comparison with magnetic tape as a standard analog data storage medium, an area of 1143 mm^2^ is needed to store a one second audio signal. Thus, in the current form, the capacity of a unit area for plasmonic film is 5600 times larger than the conventional magnetic tape.

Aside from the recording of a time-varying audio signal, we also demonstrate that the plasmonic film can be used to directly store the spectral information of an audio signal. This is achieved simply by taking the Fourier transform of the original time-domain signal and optically writing the corresponding amplitude and phase spectra on the plasmonic film. For demonstration purposes, we construct a 600-ms duration audio signal comprising of three notes (C_4_, E and G), and transfer this information into the frequency domain. Each of the notes are equally separated with a duration of 200 ms. Figures 5a and 5b are the respective amplitude and phase spectra for the transformed audio. We observe the 3 peaks in the amplitude spectrum, corresponding to the frequencies of the three recorded notes at 261.63 Hz, 329.63 Hz, and 392 Hz. The amplitude value elsewhere is close to zero and thus has negligible contribution to the retrieved signal. To get a better contrast, logarithmic plots of the amplitude spectrum are shown in Fig. 5. Based on these images, the retrieved spectra [shown in Fig. 5(e, f)] are obtained and then inverse Fourier transformed back to the time domain for audio playback (see Supplementary audio 4 and 5 for the original and retrieved audio files, respectively).

Given that the spectrum is displayed on the plasmonic film, basic signal processing can be achieved by physically ablating unwanted frequency components on the nanostructure. In this case, we design a simple filter to block two of the low-frequency components (261.3 Hz and 329.63 Hz,) on the plasmonic film, indicated by the two shaded rectangular regions (each spanning 25 Hz) in Fig. 5a. Thus, a scanning laser is used to physically ablate these two rectangular areas as shown in Fig. 5g. Note that the sharp edge of the filter is limited by diffraction only. Following the standard audio retrieval procedure, the filtered audio signal is reconstructed, with the value of the signal in the filtered region set to a null. The amplitude for the first 400 ms is close to zero. As can be heard in Supplementary audio 6, only the G note survives the filtering process, as expected.

To conclude, we present the first demonstration of audio recording and reading using reconfigurable, pillar-supported bowtie nanoantennas. We showed that an audio signal can be stored either as a time-varying waveform or as frequency spectra. Simple signal processing is demonstrated by intentionally ablating the spectra in situ. Compared with the conventional magnetic film for analog data storage, the storage capacity of pBNAs is around 5600 times larger. Currently, we are exploring combining audio and video on the pBNAs. Furthermore, by employing emerging nanomanufacturing techniques, such as nanoimprint lithography^17^, such technology could conceivably be mass produced and explored for potential data storage applications, including enhancing the niche, but still important, analog technology used in the area of archival storage (e.g., using microfiche); it also could be an important component in the development of on-chip, plasmonic-based information processing technologies^18^. Future improvements of our approach include using higher-numerical-aperture optics and pBNAs that are tuned to have a plasmonic response in the blue in order to increase the resolution in the writing; we will also explore the use of an illumination beam with a flat-top focal spot and a steep roll-off to achieve a sharper waveform edge in order to mitigate post processing^19^.

## Methods

A plasma-enhanced chemical vapor deposition (PECVD) system was used to deposit 500 nm of silicon dioxide (SiO_2_) on top of the 25-nm thick, ITO-coated, 0.4-mm thick glass substrate (CEC080P from Praezisions Glas & Optik GmbH, Germany). The ITO coating was used as a conductive layer which was needed for exposing the bowties in the e-beam lithography process. Next, the bowtie arrays were patterned by using a JOEL electron beam lithography (EBL) system. A 100 nm-thick layer of PMMA e-beam resist was spun on the substrate and baked at 200°C for 2 minutes. The resist was patterned using an e-beam acceleration voltage of 50 keV and exposed to a dose of 250 *μ*C/cm^2^. After exposure, the resist was developed in IPA:MIBK 3:1 for 45 seconds, rinsed in isopropyl alcohol for another 30 s and dried under a stream of high-purity nitrogen. For the metal evaporation process, layers of 5 nm Cr, 50 nm Au, and 8 nm Ni were deposited using an electron-beam dual gun evaporation chamber equipped with a quartz crystal monitor to measure the thickness. The reason for the Ni deposition was to protect the physical etching of gold from the reactive ion etching used to etch SiO_2_. The excess resist, Cr, Au, and Ni was removed by lift-off using an acetone bath followed by isopropyl alcohol rinse. Following the lift-off process, the substrate was dried under a stream of nitrogen to have the bowtie structure made on top of the SiO_2_. Using the bowties as a mask for RIE etching, the high aspect ratio pBNA structure was finally obtained by etching 500 nm of SiO_2_ from the bottom of the bowtie structure using a PlasmaLab reactive ion etching system with 35 mTorr pressure, 90 W power, and 70 sccm CF4 flow rate at ~25 nm/min for 20 minutes^11^,^13^,^20^.

The optical response of the exposed and unexposed film is assessed by measuring the spectral reflectance. To achieve this, a supercontinuum source is used to focus onto the sample surface of the exposed and unexposed regions (20 × 20 *μ*m^2^). The supercontinuum optical source (Femtowhite 800, NKT Photonics) is pumped using a Ti:Sapphire laser with 100-fs pulse length, 80 MHz pulse repetition rate, and 800 nm center wavelength at a power of 200 mW. The incident light from the source is coupled into the microscope (IX-81, Olympus) equipped with a 0.6-NA objective. The reflected light was collected by the same objective, and reflection spectra are taken using a fiber-coupled spectrometer (USB-2000+, Ocean Optics).

## Supplementary Material

Supplementary Information Audio 1

Supplementary Information Audio 2

Supplementary Information Audio 3

Supplementary Information Audio 4

Supplementary Information Audio 5

Supplementary Information Audio 6

## Figures and Tables

**Figure 1:**
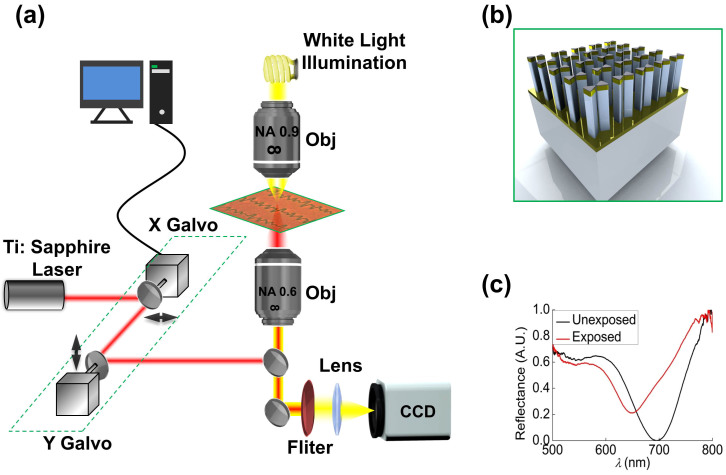
Schematic images of (a) experimental setup and (b) pBNAs. (c) Experimental spectral response of exposed and unexposed area of pBNAs showing the localized surface plasmon resonance shift.

**Figure 2:**
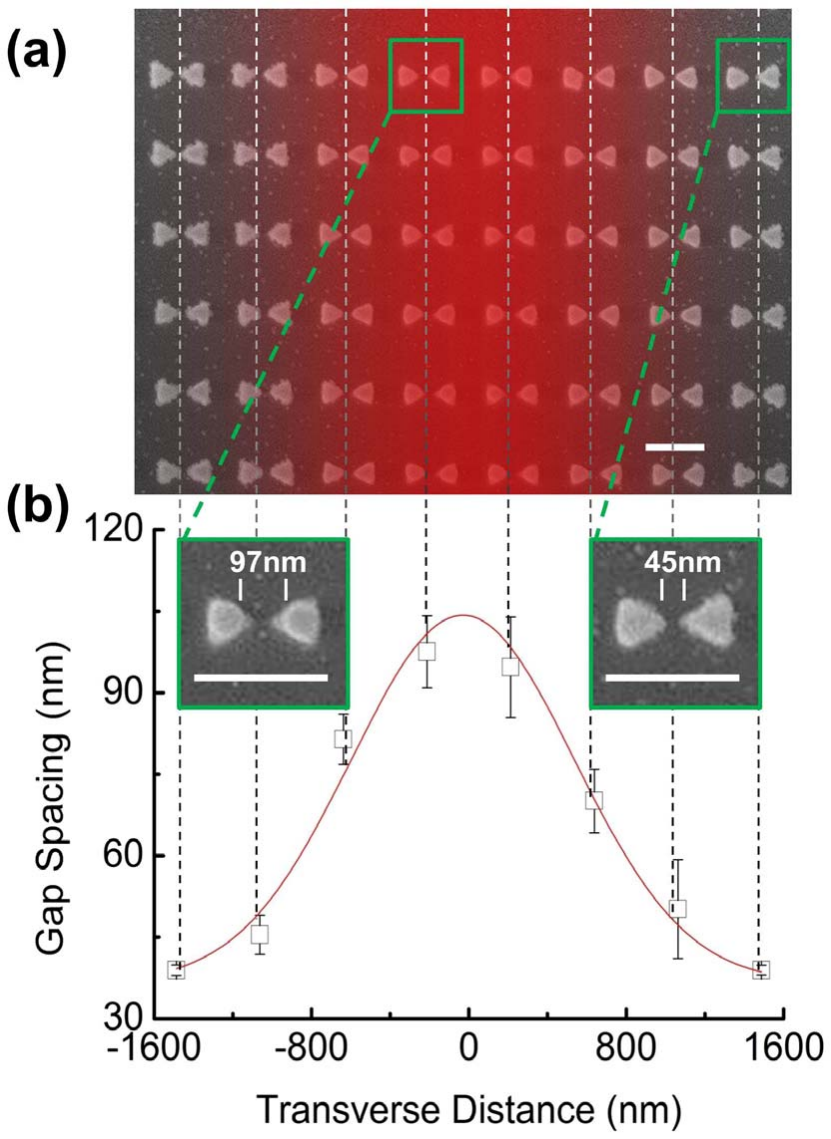
(a) Top view SEM images of an area of the pBNAs written with a Gaussian beam (shown in red over the SEM). (b) Line plot showing the change in gap spacing versus transverse position along the Gaussian beam shown in the SEM in (a). Insets from left to right are zoomed in SEM images for two representative pBNA pairs located at the center and edge, respectively. The scale bar represents 300 nm.

**Figure 3:**
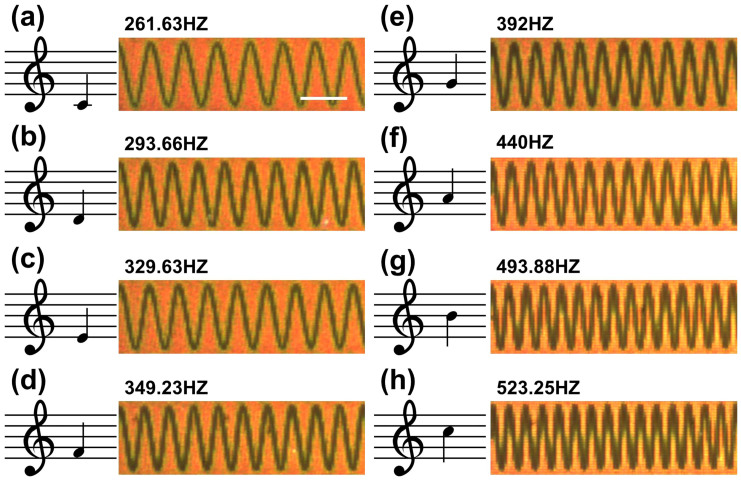
The ‘plasmonic keyboard’. Dark-field images of pBNA regions, each showing one of the 8 recorded sinusoidal notes plotted (a–h); the corresponding musical notations are shown on the left as reference. The scale bar in (a) represents 10 *μ*m and is the same for (b–h). See Supplementary audio 1 for a short song comprising the 8 recorded notes.

**Figure 4:**
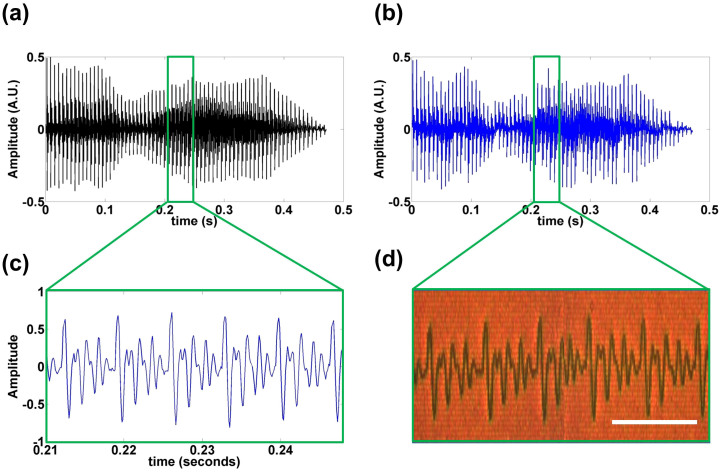
Real space audio writing. (a) Original audio data [vocal recording from author Qing Ding] and (b) retrieved signal from the pBNAs. (c) is a representative zoomed-in segment from the original audio for time range t = 0.21 s to t = 0.25 s, and (d) is an image of corresponding, recorded, time segment on a region of the pBNAs. Scale bar represent 50 *μ*m. See Supplementary audio 2 and 3 for the original and retrieved audio files, respectively.

**Figure 5:**
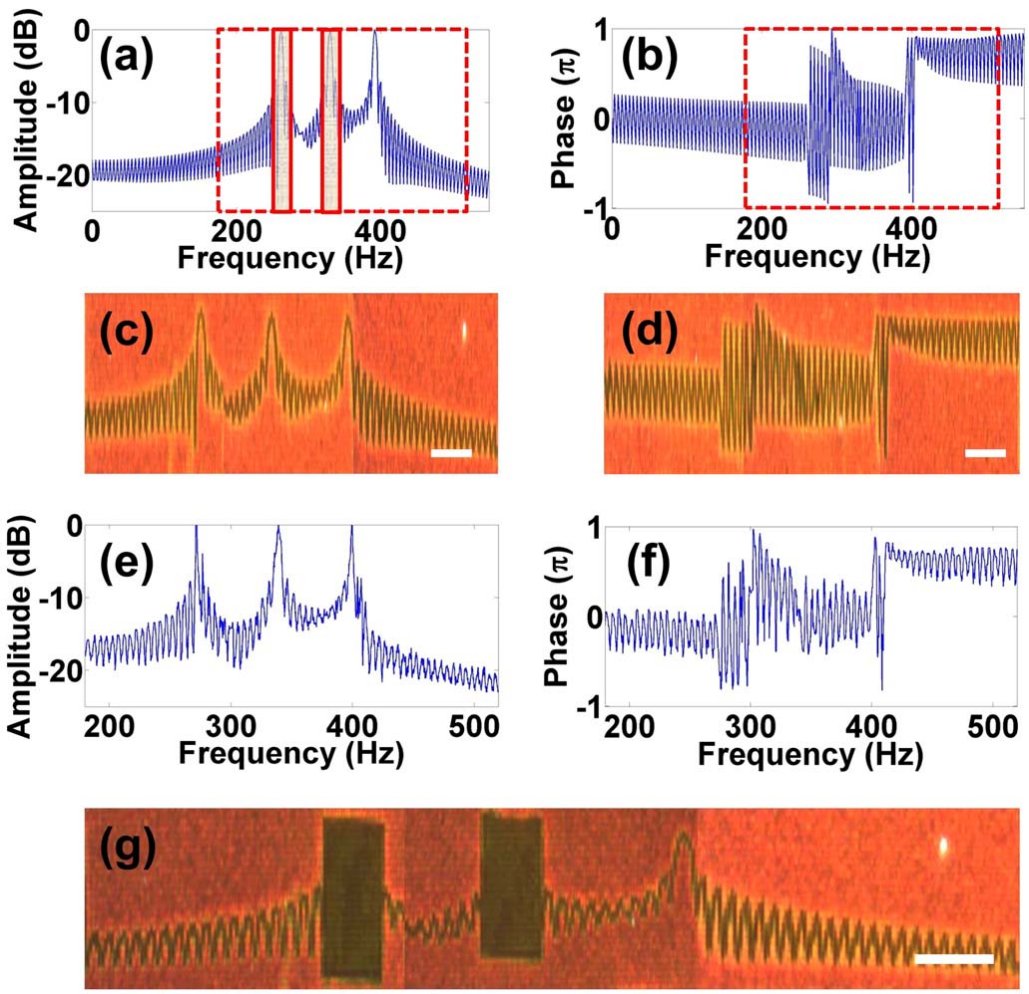
Fourier space audio writing and filtering. Original amplitude and phase spectra (a, b). The shaded rectangular regions indicate the two spectral windows, 250–275 Hz and 325–345 Hz, to be blocked. Dark-field image of recorded data from pBNAs (c, d) and corresponding retrieved amplitude and phase spectra from the recorded pattern (e, f). Recorded amplitude spectrum (g). The desired spectral windows are ablated. Scale bars represent 10 *μ*m. See Supplementary audio 4, 5 and 6.

**Table 1:** Correlation coefficient for 8 distinct notes

	C_4_	D	E	F	G	A	B	C_5_
*ρ_X,Y_*	0.995	0.994	0.990	0.983	0.985	0.983	0.974	0.932
